# 509 Relationship of Full Thickness Burn And Volume of Initial Fluid Resuscitation After Major Burn Injury

**DOI:** 10.1093/jbcr/irad045.106

**Published:** 2023-05-15

**Authors:** Arpana Jain, Claudia Islas, Samin Kabir, Dora Le, Rachna Guntu, Karen Richey, Kevin Foster

**Affiliations:** AZ Burn Center at Valleywise Health, Phoenix, Arizona; AZ Burn Center at Valleywise Health, Phoenix, Arizona; Arizona Burn Center Valleywise Health, Phoenix, Arizona; Arizona Burn Center Valleywise Health, Phoenix, Arizona; Arizona Burn Center Valleywise Health, Phoenix, Arizona; Arizona Burn Center Valleywise Health, Phoenix, Arizona; AZ Burn Center at Valleywise Health, Phoenix, Arizona; AZ Burn Center at Valleywise Health, Phoenix, Arizona; AZ Burn Center at Valleywise Health, Phoenix, Arizona; Arizona Burn Center Valleywise Health, Phoenix, Arizona; Arizona Burn Center Valleywise Health, Phoenix, Arizona; Arizona Burn Center Valleywise Health, Phoenix, Arizona; Arizona Burn Center Valleywise Health, Phoenix, Arizona; AZ Burn Center at Valleywise Health, Phoenix, Arizona; AZ Burn Center at Valleywise Health, Phoenix, Arizona; AZ Burn Center at Valleywise Health, Phoenix, Arizona; Arizona Burn Center Valleywise Health, Phoenix, Arizona; Arizona Burn Center Valleywise Health, Phoenix, Arizona; Arizona Burn Center Valleywise Health, Phoenix, Arizona; Arizona Burn Center Valleywise Health, Phoenix, Arizona; AZ Burn Center at Valleywise Health, Phoenix, Arizona; AZ Burn Center at Valleywise Health, Phoenix, Arizona; AZ Burn Center at Valleywise Health, Phoenix, Arizona; Arizona Burn Center Valleywise Health, Phoenix, Arizona; Arizona Burn Center Valleywise Health, Phoenix, Arizona; Arizona Burn Center Valleywise Health, Phoenix, Arizona; Arizona Burn Center Valleywise Health, Phoenix, Arizona; AZ Burn Center at Valleywise Health, Phoenix, Arizona

## Abstract

**Introduction:**

Full thickness burn injury affects the host response to burn injury differently that partial thickness burn injury. Current guidelines for initial resuscitation calculate fluid infusion based on Total Body Surface Area (TBSA) affected and patient’s weight. The widely used formula does not offer guidance based on the depth of the injury. We present an investigation into relationship of full thickness burn injury on fluid resuscitation. This study was approved by our Institutional Review Board.

**Methods:**

We performed a retrospective review of patients’ charts who were admitted after major burn injury of greater than 20% TBSA to a busy regional burn center. We included patients over age of 14 years admitted from year 2014-2019. We excluded patients who did not have complete resuscitation data, allowing 99 patients for analysis. We performed t test to uncover relationship of age, gender, body weight, TBSA and percent of full thickness burn with fluid received during initial resuscitation with significance set at p < 0.05.

**Results:**

The volume of fluids given during initial resuscitation was significantly affected by gender and full thickness burn injury. Fluids requirement for females was 4.6ml/kg/TBSA vs males was 3.3ml/kg/TBSA (p value 0.04). Fluid requirement for full thickness burn was 8.4ml/kg/TBSA vs 3.7ml/kg/TBSA for all patients (p=0.0004). Initial fluid resuscitation fluids did not have significant relationship with age, body weight or overall TBSA.

**Conclusions:**

This study demonstrates that full thickness burn injury has a significant impact on initial fluid resuscitation. Further research is needed to elicit the mechanism to explain the higher fluid requirement for full thickness burn injury.

**Applicability of Research to Practice:**

Our study brings attention to the effect of depth of burn injury on the actual volume of fluids needed during initial resuscitation of burn injury. This study will alert the clinician to take into account both the surface area as well as depth of the burn injury while embarking on fluid resuscitation after a major burn injury.

